# New microsatellite markers for population studies of *Phytophthora cinnamomi*, an important global pathogen

**DOI:** 10.1038/s41598-017-17799-9

**Published:** 2017-12-15

**Authors:** J. Engelbrecht, T. A. Duong, N. v. d. Berg

**Affiliations:** 1 Department of Microbiology, Forestry and Agricultural Biotechnology Institute (FABI), University of Pretoria, Pretoria, 0002 South Africa; 2 Department of Genetics, Forestry and Agricultural Biotechnology Institute (FABI), University of Pretoria, Pretoria, 0002 South Africa

## Abstract

*Phytophthora cinnamomi* is the causal agent of root rot, canker and dieback of thousands of plant species around the globe. This oomycete not only causes severe economic losses but also threatens natural ecosystems. In South Africa, *P. cinnamomi* affects eucalyptus, avocado, macadamia and indigenous fynbos. Despite being one of the most important plant pathogens with a global distribution, little information is available regarding origin, invasion history and population biology. This is partly due to the limited number of molecular markers available for studying *P. cinnamomi*. Using available genome sequences for three isolates of *P. cinnamomi*, sixteen polymorphic microsatellite markers were developed as a set of multiplexable markers for both PCR and Gene Scan assays. The application of these markers on *P. cinnamomi* populations from avocado production areas in South Africa revealed that they were all polymorphic in these populations. The markers developed in this study represent a valuable resource for studying the population biology and movement of *P. cinnamomi* and will aid in the understanding of the origin and invasion history of this important species.

## Introduction

*Phytophthora cinnamomi* (Rands), causal agent of Phytophthora root rot, affects close to 5000 plant species across the globe^1–3^ including important native species in Australia and South Africa and perennial trees such as avocado, macadamia, peach, oak and chestnut^4^. Over the last decade, this destructive pathogen has caused several global epidemics, including root rot of chestnut in America and jarrah dieback in Australia. This destructive pathogen has been included in the list of the 10 most destructive oomycetes^5^ and as one of the 100 worst invasive species worldwide by Invasive Species Specialist Group (ISSG: http://www.issg.org).

*Phytophthora cinnamomi* was first isolated from stripe cankers of cinnamon trees in Sumatra^6^ and has since been reported from various countries around the globe. The centre of origin remains a controversial topic with authors speculating that it originated from South-East Asia as it seems to be indigenous to tropical and subtropical countries. Indonesia, Malaysia, Taiwan and New Guinea have all been proposed as the centre of origin of this species^7–9^. South Africa has also been proposed as having indigenous *P. cinnamomi* populations^10^.

*Phytophthora cinnamomi* has a broad host range and disease symptoms vary significantly between tree species. Infection usually occurs through the feeder roots, causing root rot that eventually progresses into larger roots. It can also infect root crowns, trunks, and woody stems forming a reddish-brown canker^1^. Above-ground symptoms include leaf chlorosis, wilting and dieback.

*Phytophthora cinnamomi* is a heterothallic species which requires the presence of both A1 and A2 mating types to undergo sexual reproduction. Both mating types of the pathogen have been reported to be present in natural ecosystems such as in Australia and South Africa^11–13^. However, the presence of both mating types in natural populations does not infer that sexual reproduction is occurring^14^. Asexual reproduction takes place through the formation of large numbers of sporangia, that release motile zoospores^4,15^. This is believed to be the predominant mode of reproduction and spread of the pathogen in natural populations.

Genetic variability of *P. cinnamomi* has been studied using various markers including isozymes, RAPD, AFLP, microsatellites, mitochondrial haplotypes, and direct gene sequencing^11,12,15–21^. In all of these studies, *P. cinnamomi* was found to have low genetic variability within and among geographically defined populations. Interestingly, the A1 and A2 mating types can often be distinguished based on the genetic differentiation^19,22^.

Microsatellite (or simple sequence repeats - SSR) markers are short tandem repeats found in abundance and randomly distributed across the genomes of eukaryotic organisms^23,24^. The ease of amplification and high level of polymorphism observed in these markers have made them the marker of choice for many studies^25,26^. Prior to the genome sequencing era, the development of SSR markers posed several challenges as this process required prior knowledge of SSR regions and its flanking sequences for primer design. This, however, has changed as whole genome sequences are now readily available or can be generated at a reduced cost. The development of a good set of SSR markers, however, still requires screening a large number of markers on a sufficient number of individuals to search for polymorphisms, which ultimately still involves considerable cost and effort.

Although *P. cinnamomi* is an organism of ecological and economic importance, there are currently only three microsatellite markers available for this organism^17^. Here we describe the development and validation of a set of 16 polymorphic SSR markers which can be used for studying population diversity and movement of *P. cinnamomi* in natural populations as well as commercial orchards and plantations. Two populations of *P. cinnamomi* from commercial avocado orchards in South Africa were used to assess the informativeness of these markers.

## Materials and Methods

### Isolate sampling, identification and DNA extraction

Avocado roots and soil samples surrounding avocado trees were collected from Limpopo and Mpumalanga provinces in South Africa. Direct isolations were made by embedding root pieces in PARPH media (20% clarified V8 juice agar containing 0.02 g of pimaricin, 0.25 g of ampicillin, 0.01 g of rifampicin, 0.10 g of PCNB, and 0.075 g of hymexazole). Isolations from soil were made by baiting with avocado leaves followed by plating the leaves onto PARPH media.

A total of 211 isolates (90 from Limpopo and 121 from Mpumalanga) were confirmed to be *P. cinnamomi* using morphological features. Single hyphal tip cultures were made and subsequently inoculated into malt-yeast extract medium (2% malt extract, 0.5% yeast extract) and grown for four days at 25 °C @ 200 rpm. Mycelia were harvested, freeze dried and genomic DNA was extracted using the PrepMan^®^ Ultra Sample Preparation Reagent (Applied Biosystems, USA) as described previously^27^.

### Identification of polymorphic microsatellite regions

Genome data from three isolates of *P. cinnamomi* were used in the development of SSR markers. These included a publically available genome from the Joint Genome Institute (JGI) (http://genome.jgi.doe.gov/Phyci1/Phyci1.home.html) and two recently published genomes from New Zealand and Australia^28^. The assembled genome (v. 1.0) of *P. cinnamomi* obtained from JGI was used to search for regions containing microsatellite repeats using the REPET package^29^, which combines SSR identification from TRF^30^, Mreps^31^ and RepeatMasker^32^. The genomic locations of SSR repeats were extracted from the REPET annotation result and used to create a SSR region file to be used for genotyping using RepeatSeq^33^.

**Figure 1:**
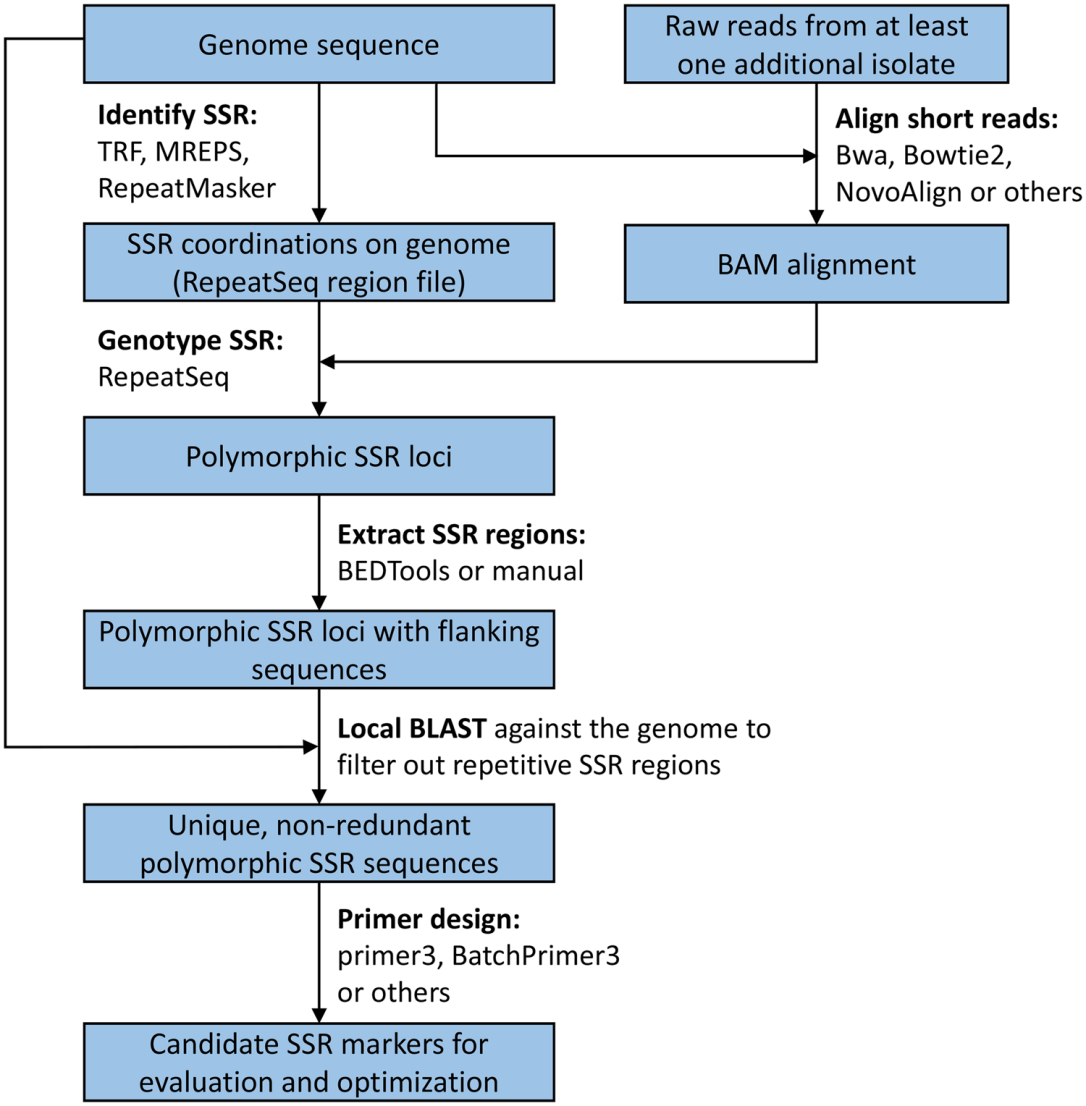
Schematic illustration of the SSR marker development pipeline used in this study.

Whole genome genotyping of SSR repeats was done using RepeatSeq v0.8.2^33^. To do this, paired-end Illumina reads from two other *P. cinnamomi* isolates from Australia and New Zealand^28^ were downloaded from Sequence Read Archive (SRA), trimmed and aligned to the reference genome (JGI assembled *P. cinnamomi* genome used for SSR identification) with Bowtie2^34^ using a local-sensitive option. The BAM files (one file for each isolate) were used for genotyping of SSR repeats using RepeatSeq with the SSR region files created in the previous step. Polymorphic SSR regions between the reference and the genotyped isolates were identified from the RepetSeq output. The workflow used for SSR identification and marker development is summarized in Fig. 1.

### SSR marker design

In this study, we only targeted tri- and tetra-repeat SSRs that were found to be polymorphic between the reference genome and any of the two genotyped isolates for marker development. The polymorphic SSR regions as well as their 500 bp upstream and downstream flanking sequences were extracted from the reference genome. These sequences were then subjected to a local BLAST analysis against the assembled genome and any sequence that showed multiple homologies to different regions in the genome was removed. Only candidate sequences with a single unique location in the genome were further selected for marker development. The program BatchPrimer3^35^ was used to designed primers flanking the SSR regions. Primer pairs were selected based on their compatibilities for PCR and genescan multiplex assays (similar primer annealing temperatures and wide distribution of expected amplicon sizes) and genomic locations of the markers (avoiding markers located in close proximity on a large scaffold and to avoid more than one marker on a small scaffold).

PCR for evaluation of selected markers consisted of 1X PCR reaction buffer, 2.5 mM MgCl_2_, 200 µM of each dNTPs, 0.2 µM of each primer, 1 U FastStart *Taq* DNA Polymerase (Roche Applied Science, Germany), and 20 ng of genomic DNA, in a total volume of 25 µL. Amplification was performed with the following conditions: initial denaturation at 95 °C for 5 min, followed by 35 cycles of 94 °C for 30 s, 58 °C annealing for 30 s, and 72 °C extension for 60 s, and a final extension at 72 °C for 30 min. PCR products were visualized on 1.5% agarose gels and stained with GelRed (Biotium, Hayward, California, USA) to assess primer specificity. Products obtained were sequenced to confirm that the primers amplified the targeted SSR regions.

### Multiplex PCR optimization and SSR genotyping

In total, 16 primer pairs were selected to make up a single panel of SSR markers for *P. cinnamomi* (Table 1). Based on their expected amplicon sizes, these markers were labelled with four different dyes (FAM, PET, VIC and NED) in such a way that would allow combined genotyping of all markers in a single GeneScan run per isolate. Initially, 16 markers were separated into two separate multiplex PCRs: multiplex-1 included Pc_SSR2, Pc_SSR4, Pc_SSR14, Pc_SSR15, Pc_SSR17, Pc_SSR19, Pc_SSR20 and Pc_SSR23; and multiplex-2 included Pc_SSR3, Pc_SSR7, Pc_SSR8, Pc_SSR9, Pc_SSR12, Pc_SSR13, Pc_SSR16 and Pc_SSR22. Multiplex PCR amplification was carried out using the Platinum® Multiplex PCR master mix (Applied Biosystems). Each of the amplification reactions consisted of 1X PCR master mix, forward and reverse primers for the respective multiplex with primer concentrations as indicated in Table 1, 20 to 50 ng of template DNA, and PCR-grade water to a total volume of 25 µL. The thermal cycle conditions were as follows: initial denaturation at 95 °C for 2 min, followed by 35 cycles of 95 °C for 30 s, 58 °C for 30 s, and 72 °C for 60 s, and a final extension at 72 °C for 30 min. PCR products from both multiplex-1 and multiplex-2 were diluted 25 times and 1 µL of diluted product was mixed with 8.8 µL formamide and 0.15 µL GeneScan LIZ-500 standard (Applied Biosystems), denatured at 95 °C for 5 min before fragments were separated using an ABI PRISM 3100 Genetic Analyzer (Applied Biosystems). Allele sizes were determined using GENEMAPPER v4.0 (Applied Biosystems). Initially, PCR and GeneScan were conducted separately for multiplex-1 and multiplex-2 per isolate. However, once the expected alelle sizes for the markers have been pre-determined using a sub-set of the population, all 16 markers can then be combined into a single multiplex PCR and GeneScan run for each isolate.

**Table 1:** Information on 16 SSR markers for *Phytophthora cinnamomi* including repeat motifs, labelling dyes, primer sequences, annealing temperature (Ta), primer concentrations to be used in multiplex PCRs, number and sizes of observed alleles, and GenBank accessions of representative alleles.

Name	Repeats	Dye	Primer sequences 5′ to 3′	Ta (°C)	Multiplex	Primer Concentration	Observed alleles (bp)	GenBank Asscession
Pc_SSR19	(CTG) n	6-FAM	F: CTACTGGAACGTGCTGAACC R: GGCACAGGCACCTTGAAC	58	Multiplex-1	0.2 µM	171, 174	MG436982, MG436983
Pc_SSR4	(CAG) n	NED	F: GGCAGCTTCATACTGGAATCAA R: CTTGGCCTTGCGGATGGA	58	Multiplex-1	0.2 µM	173, 176	MG436958, MG436959
Pc_SSR14	(TTC) n	VIC	F: AACATTTGCTGCTACTGACGAT R: TACCACCACTAGAAGTCCAGAG	58	Multiplex-2	0.1 µM	212, 215	MG436972, MG436973
Pc_SSR12	(CTG) n	6-FAM	F: CGGAATGAATGGCGTGGAG R: GCGGCTGCTGGTCAAGTC	58	Multiplex-2	0.2 µM	211, 214	MG436967, MG436968
Pc_SSR8	(GCT) n	NED	F: CAGCGGCATCAGCAACAC R: GCGAAGCGATGGACAATGG	58	Multiplex-2	0.2 µM	211, 217, 220	MG436962, MG436963, MG436964
Pc_SSR7	(GAGG) n	PET	F: ATAGCTGGCGTCAGGATGG R: CATGTTTGCTCGGTTGAATACG	58	Multiplex-2	0.2 µM	262, 268	MG436960, MG436961
Pc_SSR22	(GCT) n	VIC	F: CGTATCCGTGGCTGTGATG R: GGAGACATGGGCATGATGG	58	Multiplex-2	0.2 µM	253, 256, 262	MG436986, MG436987, MG436988
Pc_SSR2	(CAG) n	6-FAM	F: GGAGTGTGCTGCGTGTGA R: CGAGTCGGAGTAGTCGTCAA	58	Multiplex-1	0.1 µM	266, 269	MG436954, MG436955
Pc_SSR20	(CAG) n	NED	F: GGAGGTCCAGAGACTGTGG R: CTTGAGGTGCGGCGAGAT	58	Multiplex-1	0.2 µM	316, 319	MG436984, MG436985
Pc_SSR15	(ATT) n	PET	F: GAGTCGTGTTCGTTGCCTTT R: CGTCTTGAAGTTGATGCTGCTA	58	Multiplex-1	0.1 µM	320, 326	MG436974, MG436975
Pc_SSR23	(GTG) n	VIC	F: CACGGTGGTGAACAATGACA R: AACGACTGCTGGATAGGAACA	58	Multiplex-1	0.2 µM	333, 336, 339	MG436989, MG436990, MG436991
Pc_SSR16	(TGCC) n	6-FAM	F: CTTGCCACCTGATACCACATC R: GAGCGGCGACTACGACTA	58	Multiplex-2	0.2 µM	339, 343	MG436976, MG436977
Pc_SSR9	(AGC) n	NED	F: CCGCATCATCACTTGAAACG R: CTACGCCCAGACACAGACA	58	Multiplex-2	0.1 µM	350, 353	MG436965, MG436966
Pc_SSR3	(CAA) n	PET	F: TGGTAGTGTTGTGTTCGTGAG R: CTGCGTCGTGAAGCCATG	58	Multiplex-2	0.4 µM	377, 380	MG436956, MG436957
Pc_SSR13	(CTT) n	VIC	F: CTCCACCTCGAACTGCTTGT R: CTCGTCGTGCTGCGTCTG	58	Multiplex-2	0.2 µM	437, 443, 458	MG436969, MG436970, MG436971
Pc_SSR17	(CTG) n	6-FAM	F: GAAGACGGTGCGGAAGCT R: GCCATTAGCCAAACGAGTCC	58	Multiplex-1	0.2 µM	424, 430, 433, 436	MG436978, MG436979, MG436980, MG436981

### Evaluation of markers informativeness on natural populations of *P. cinnamomi*

A collection of 211 *P. cinnamomi* isolates from two different geographical locations in South Africa were used to test the informativeness of the markers. For the first randomly selected 60 isolates, two multiplex PCRs and GeneScan runs were conducted separately for multiplex-1 and multiplex-2 as described above. The initial results indicated that the observed allele sizes did not overlap in these isolates, and thus single multiplex PCRs of all 16 markers were subsequently conducted for each of the remaining isolates. PCR mixture, primer concentrations, thermal conditions, and GeneScan were the same as described in the previous section.

### Confirmation of allele sizes

All observed alleles for all 16 markers were confirmed with traditional Sanger sequencing. To do this, isolates representing various allele sizes were selected and PCRs were conducted separately for each of the markers using non-labelled primers (PCR mixtures and conditions were the same as previously described). PCR products of homozygous alleles were sequenced directly whereas PCR products of heterozygous alleles were cloned using CloneJET PCR cloning kit (Thermo Scientific, USA), followed by colony PCR amplification and sequencing of the PCR products. Sequences obtained from sequencing with forward and reverse primers were assembled for each allele, and the exact allele size was determined directly from the sequence. We used the exact allele sizes determined by sequencing to correct the sizes obtained from GeneScan analysis. Sequence data for each of the observed alleles from all markers were deposited into GenBank for future reference - GenBank accession numbers are indicated in Table 1.

### Genetic diversity, population differentiation and linkage disequilibrium

The R package *poppr* ^36^ was used to calculate basic population statistics, including (i) Stoddart and Taylor’s index, *G* = *1*/Σ_*i*_ *p* ^2^_*i*_, where *p*_*i*_ is the observed frequency of *i* ^*th*^ genotype^37^; (ii) evenness, *E*_5_ = (*1/*λ) − *1/e* ^*H′*^ − *1*, where λ is Simpson’s index and H′ is Shannon-Wiener’s index; and (iii) Bruvo’s distance^38^. E_5_ is a preferred index of evenness because it is less dependent on the number of genotypes in a sample^39^. Allele frequency at each locus and F statistics (F_ST_) were calculated using GENALEX v 6.5^40^. Fixation index F_ST_ was used to examine the overall genetic differentiation between populations.

The hypothesis of random mating within populations was tested using the index of association statistics. The rBarD value, which corresponds to the index of association (I_A_) but is independent from the number of loci considered, was calculated with Multilocus v1.2^41^. The observed rBarD values were calculated for each population and compared to that obtained from a 1000-time randomized simulation.

### Inference of population structure

Principal component analysis (PCA) based on the genetic distance metric of Bruvo^38^ was conducted using the R package POLYSAT v1.3.3^42^. Since POLYSAT supports polyploidy data; all markers were included in the PCA analysis. STRUCTURE 2.3 was used to infer and assign population structure^43^. The MCMC estimation was conducted for 5 million generations, with an initial burn-in of 20 000, K ranging from 2 to 5 with 20 iterations for each K. The program CLUMPAK^44^ was used to average and cluster Structure results for each K using the Markov clustering algorithm (MCL) with default settings. Since STRUCTURE does not support polyploidy data, for makers with more than two alleles per locus, the last alleles were removed prior to analysis. Minimum spanning networks (MSN) using Bruvo’s distance was constructed using R package *poppr* ^36^ to show possible evolutionary relationships among multilocus genotypes (MLGs).

## Results

### SSR identification, primer design and PCR amplification

Analysis of the *P. cinnamomi* genome using REPET *de novo* pipeline identified a total of 39,141 SSR regions, 7077 of which were tri- and 3332 were tetra-repeats. Genotyping of these tri- and tetra-repeat regions on two additional available genomes using RepeatSeq^33^ revealed that 165 of the tri-repeats and 62 tetra-repeats were polymorphic between the reference and the genotyped isolates. Further filtering by manual curation of the repeats to remove imperfect and compound repeats resulted in 122 regions that could be considered for marker development.

After conducting a local BLAST analysis of the 122 candidate SSR regions against the genome of *P. cinnamomi* to further remove SSR regions of high complexity (having cross homology to other regions in the genome), 40 SSR regions were finally selected for primer design, 23 of these resulted in primers that met the required criteria: product size equal or less than 500 bp and primers with properties that are compatible for multiplex PCRs in downstream applications. PCR validation of the initial 23 markers resulted in 16 markers that specifically and consistently amplified the desired regions on a subset of *P. cinnamomi* isolates. These 16 markers with their primer sequences and their properties are presented in Table 1.

### Multiplexing PCR optimization

**Figure 2:**
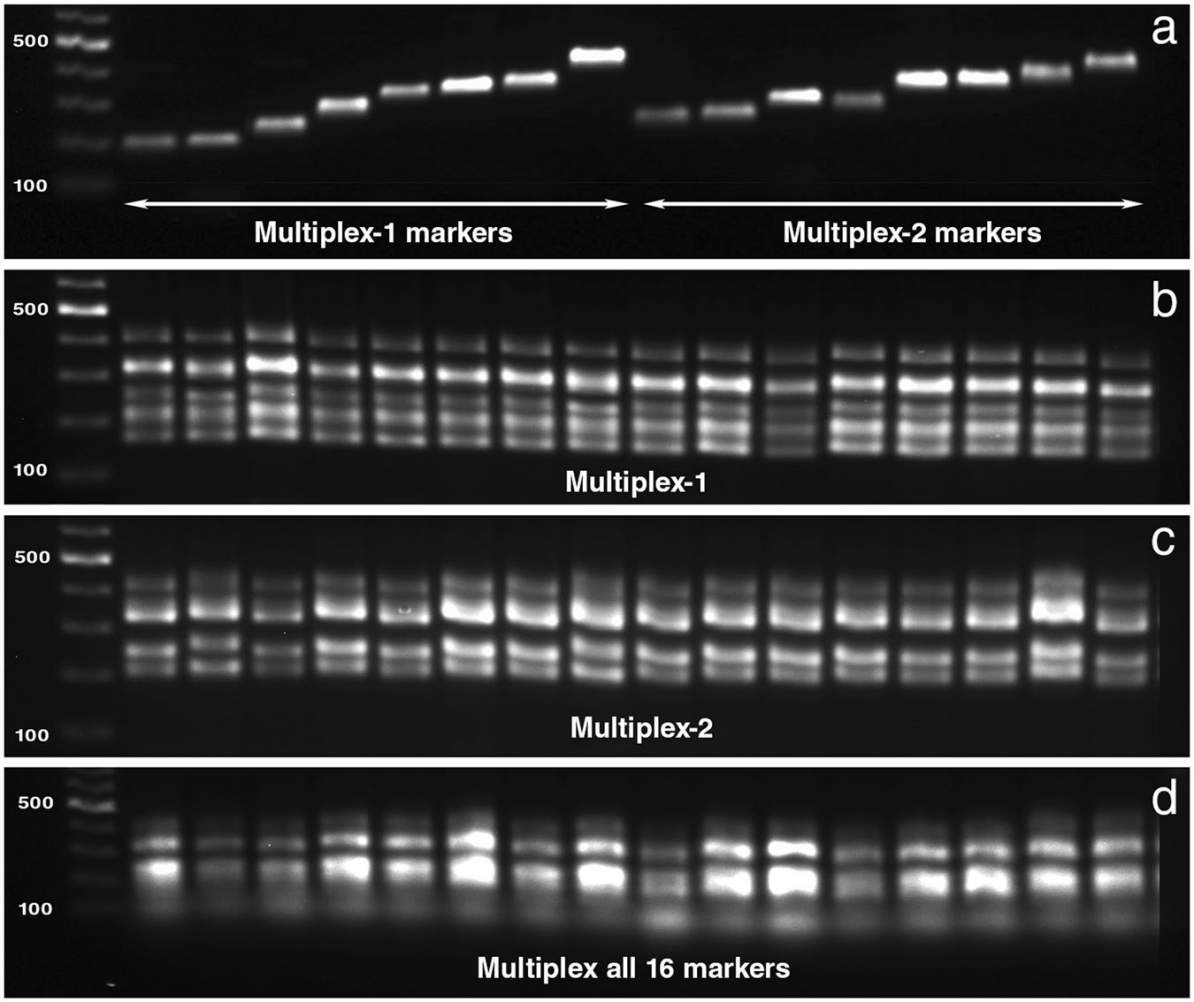
Agarose gel electrophoresis of PCR products obtained from (**a**) 16 individual SSR markers (**b**) multiplex-1 (**c**) multiplex-2 (**d**) multiplex of all 16 markers. This figure has been cropped and edited for illustration purposes – the original images are presented in the Supplementary Fig. S1. Additionally, electropherograms obtained from genescan analysis of all 16 markers multiplexed together are presented in the Supplementary Fig. S2.

Based on the allele size distribution and labelled dyes, 16 markers were divided into two separate multiplex PCRs namely multiplex-1 and multiplex-2 (Table 1 and Fig. 2). Primer concentrations for multiplex PCRs were optimised based on the intensity of the individual PCR products, and further refined based on the signals obtained from GeneScan analysis. The optimal primer concentrations for each marker to be used in multiplex PCRs is presented in Table 1. Multiplexing of all 16 markers into a single PCR and GeneScan gave identical results when compared to that obtained from two separate multiplex PCRs (multiplex-1 and multiplex-2) (Fig. 2).

### SSR genotyping of two *P. cinnamomi* populations from South Africa

The 211 isolates considered in this study were grouped into two populations according to their geographic origin. A total of 121 isolates originated from Mpumalanga and 90 isolates from Limpopo, hereafter referred to as Mpumalanga and Limpopo populations, respectively. Applying these markers on 211 *P. cinna*momi isolates revealed that all 16 markers were polymorphic in both populations. Thirteen markers produced a maximum of two alleles per isolate. Three of the markers produced three alleles in a number of isolates (Pc_SSR13 in 31 isolates, Pc_SSR22 in 134 isolates and Pc_SSR23 in 19 isolates). In total, 50 multilocus genotypes (MLG’s) were detected among 211 isolates from South Africa, 34 of which were represented by only a single isolate and nine MLG’s that were shared between the two populations. In total, 34 alleles were detected with 15 markers (Pc_SSR17 was excluded from this analysis, see below), with the number of alleles ranging from 2 to 3 alleles per locus (Tables 1 and 2). A representative isolate from each MLG was selected for PCR amplification and sequencing of the ITS region using ITS1 and ITS4 primers^45^. Sequencing results confirmed that all of the selected isolates represented *P. cinnamomi*.

**Table 2:** Allele frequency for *Phytophthora cinnamomi* populations from Mpumalanga and Limpopo, South Africa.

Locus	Allele size	Mpumalanga	Limpopo
Pc_SSR2	266	0.591	0.622
	269	0.409	0.378
Pc_SSR3	377	0.079	0.117
	380	0.921	0.883
Pc_SSR4	173	0.921	0.889
	176	0.079	0.111
Pc_SSR7	262	0.917	0.872
	268	0.083	0.128
Pc_SSR8	211	0.004	—
	217	0.426	0.389
	220	0.570	0.611
Pc_SSR9	350	0.079	0.117
	353	0.921	0.883
Pc_SSR12	211	0.580	0.618
	214	0.420	0.382
Pc_SSR13	437	0.496	0.478
	443	0.500	0.500
	458	0.004	0.022
Pc_SSR14	212	0.492	0.500
	215	0.508	0.500
Pc_SSR15	320	0.038	0.079
	326	0.962	0.921
Pc_SSR16	339	0.517	0.511
	343	0.483	0.489
Pc_SSR19	171	0.421	0.400
	174	0.579	0.600
Pc_SSR20	316	0.579	0.617
	319	0.421	0.383
Pc_SSR22	253	0.453	0.388
	256	0.458	0.461
	262	0.089	0.152
Pc_SSR23	333	0.087	0.094
	336	0.459	0.478
	339	0.455	0.428

A single dominant genotype was present in both Limpopo and Mpumalanga populations which accounted for 59% and 55% in each population, respectively. The Mpumalanga population had 32 MLGs in total where the Limpopo had 27 MLGs. Shannon-Wiener’s index (H) was similar for the two populations (Table 3). The G values were 2.81 for Tzaneen and 3.22 for Mpumalanga, and 3.06 for South Africa as a whole (Table 3). The unbiased gene diversity (Hexp) was very similar between Limpopo and Mpumalanga as well as the whole South Africa combined (Table 3).

**Table 3:** Genotypic diversity statistics for *Phytophthora cinnamomi* isolates from South Africa.

Population	N	MLG	eMLG	H	G	lambda	E.5	Hexp	Ia	rbarD	P.rD
Mpumalanga	121	32	26.20	3.47	3.22	0.69	0.31	0.44	6.35	0.469	0.001
Limpopo	90	27	27.00	3.30	2.81	0.64	0.30	0.45	5.67	0.451	0.001
South Africa	211	50	50	3.91	3.06	0.67	0.25	0.40	5.67	0.422	0.001

**N** = Number of individuals observed, **ML** **G** = Number of multilocus genotypes (MLG) observed, **eMLG** = The number of expected MLG at the smallest sample size ≥ 10 based on rarefaction, **H = **Shannon-Wiener Index of MLG diversity^57^, **G = **Stoddart and Taylor’s Index of MLG diversity^37^, **Lambda** = Simpson’s Index^58^, **E.5** = Evenness, (*E*_*5*_)^39^, **Hexp** = Nei’s unbiased gene diversity^59^, **Ia** = The index of association *I*_*A*_^60,61^, **rbarD** = The standardized index of association $\bar{{\rm{r}}}$_d_, **p.rD** = p-value for $\bar{{\rm{r}}}$_d_.

Sequencing of all representative alleles confirmed that all markers were polymorphic and the size variation observed were due to variations in the repeat units. However, the actual allele sizes as confirmed by sequencing showed variations when compared to the sizes obtained from GeneScan analysis. In particular, the actual size for Pc-SSR3 obtained from sequencing was three base-pairs longer (+3) than that obtained from GeneScan. Similarly, the values for other markers are: Pc_SSR2 (+0), Pc_SSR4 (+1), Pc_SSR7 (−2), Pc_SSR8 (−2), Pc_SSR9 (+5), Pc_SSR12 (−1), Pc_SSR13 (+1), Pc_SSR14 (−3), Pc_SSR15 (−2), Pc_SSR16 (+2), Pc_SSR17 (+3), Pc_SSR19 (+1), Pc_SSR20 (+2), Pc_SSR22 (+1), Pc_SSR23 (+1). The allele sizes presented in this study are the actual adjusted sizes as obtained from sequencing results. Marker Pc_SSR17 had two variable microsatellite regions (both consisting of tri-repeat units) and thus could lead to inaccurate estimation of the number of alleles if based solely on size variation. Therefore, this marker was excluded from further analysis. This marker was, however, highly variable and can be used if alleles are to be determined by sequencing, but not by GeneScan analysis.

### Clonality in *P. cinnamomi*

Performing index of association tests on *P. cinnamomi* isolates from Limpopo and Mpumalanga rejected the null hypothesis of random association between alleles at different loci (P < 0.001), supporting a clonal mode of reproduction. Observed I_A_ and rBarD values for clone corrected data are presented in Table 3. The observed rBarD values fell beyond the range that were obtained from the 1000 randomized dataset with significant *P* values.

### Population structure of *P. cinnamomi*

**Figure 3:**
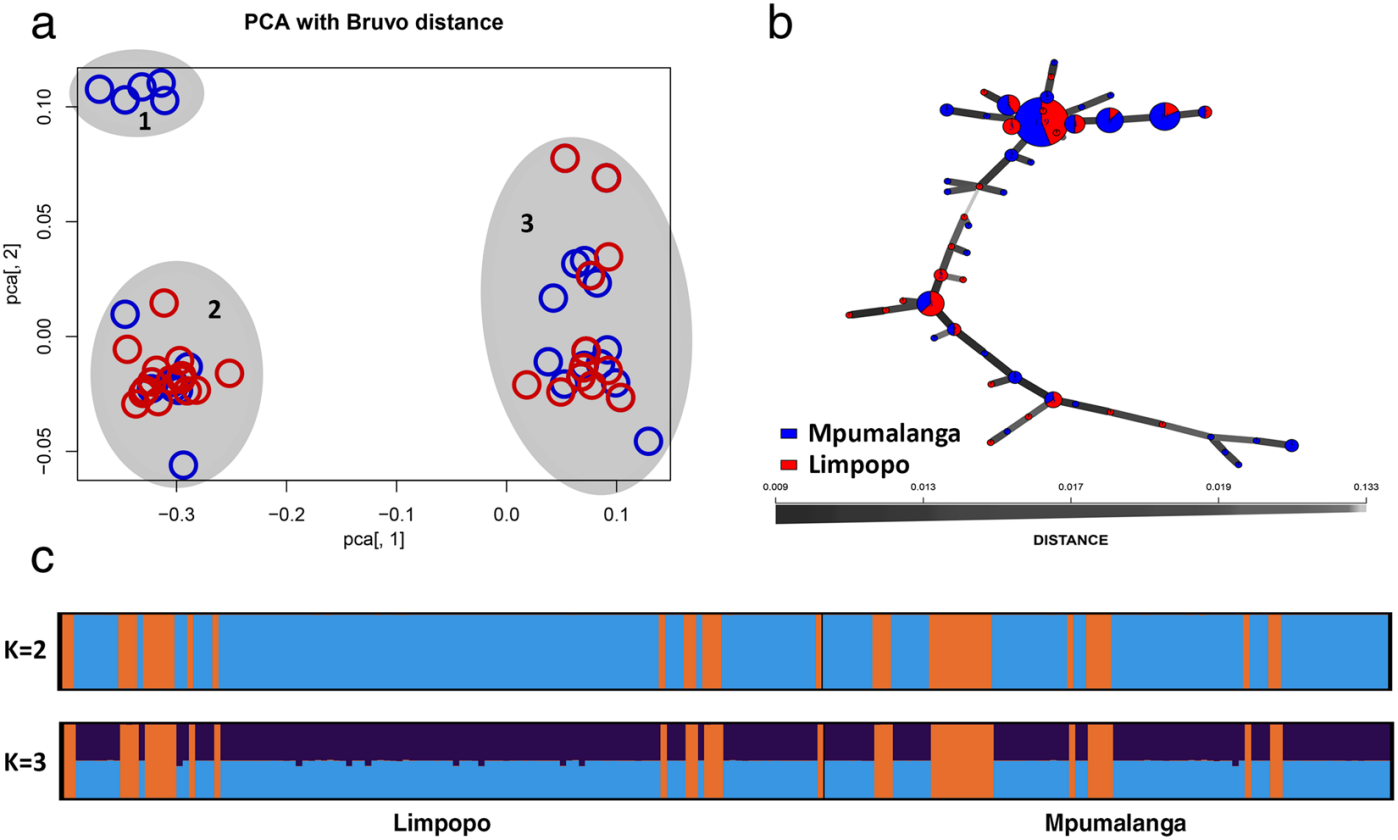
Population structure of *Phytophthora cinnamomi* isolates from South Africa (**a**) PCA analysis using Bruvo’s distances of *P. cinnamomi* isolates. The sources of isolates are indicated in red circles for Limpopo and blue circles for Mpumalanga (**b**) Minimum spanning network using Bruvo’s distances. The size of the nodes is proportional to the number of represented clones and the thickness of the lines represent the Bruvo genetic distance between two nodes (thicker lines denote smaller genetic distance) (**c**) Bar chart displaying the membership coefficients (based on Structure) of *P. cinnamomi* isolates used in this study for K = 2 and K = 3. Sources of isolates are indicated below the chart.

PCA using Bruvo’s distance divided isolates from Limpopo and Mpumalanga into three clusters, however, these were not based on the geographical origin of isolates (Fig. 3a). The dominant MLG from both the Mpumalanga and Limpopo populations resided within Cluster 3. The minimum spanning network (MSN) clearly differentiated two groups of isolates (Fig. 3b). Structure analysis assigned isolates into two distinct genetic groups (K = 2), this was also not based on geographic location. No further divisions were observed with K > 2 (Fig. 3c).

## Discussion

We have successfully developed 16 polymorphic microsatellite markers for *P. cinnamomi* using the publically available genome sequences from three *P. cinnamomi* isolates. Most importantly, these markers can be multiplexed into a single PCR and GeneScan run, which significantly reduces genotyping costs. These markers represent a valuable resource for future population genetic studies of *P. cinnamomi*. To our knowledge this is the first study to develop a comprehensive set of SSR markers for *P. cinnamomi*, apart from the three markers previously developed^17^.

The availability of whole genome sequences provides unique opportunities to identify and develop SSR markers. With the traditional approach, regions containing microsatellite markers are searched for in the genome sequence after which a number of primers are designed, followed by testing a subset of isolates to search for polymorphic markers. While this approach has been successfully used to develop markers for a number of species^46,47^, it is still time consuming and requires a significant cost in synthesizing a large number of primers and testing them for polymorphism. In this work, we described a different approach that takes advantage of the availability of multiple genome sequences to search for SSR markers that are known to be polymorphic before proceeding to primer design and marker development. Not only does this reduce the number of markers for design and testing, but it also allows attention to be focused on designing a panel of markers that better reflects genome-wide sampling and is compatible for multiplex genotyping. Gagnon, *et al*.^48^ also used sequence data from multiple *Phytophthora ramorum* isolates to identify variable sites for SSR development. Their approach is somewhat similar to ours, but did not make use of tools that are specifically developed for SSR identification and genotyping. The workflow presented in this study is more comprehensive and optimised for polymorphic SSR marker identification. With the continued cost reduction for whole genome sequencing, we believe that our approach is more cost effective and the better choice for future SSR marker development projects.

The current knowledge on genetic diversity and movement of *P. cinnamomi* in natural populations is still very limited. To date, several population studies have been conducted using isozyme analysis^11,12^, AFLP^22^, or only three SSR markers^16^. Although the use of isozyme and AFLP have been intensively used in the past and have proven to be useful in assessing diversity and relatedness, the results obtained from these techniques are often difficult to consistently reproduce and hence prohibit the transferability of the data between studies. By applying the 16 SSR markers developed in this study on various future population studies of *P. cinnamomi*, we will be able to gain a better understanding of its diversity and movement between populations, countries and continents. It is important to note that the allele sizes obtained from GeneScan will vary between different laboratories, machines, or running conditions where different size standards are being used. Thus, we highly recommended that the actual allele sizes should be confirmed by sequencing of representative alleles and use them to adjust the data obtained from GeneScan. This will facilitate the synergy of research by combining and comparing results from different studies.

Three of the 16 markers in this study had three alleles (i.e. trisomy) in a number of isolates tested. This phenomenon has also been previously reported for *P. cinnanomi* using SSR markers^17^ where a large proportion of non-Mendelian inheritance was observed. The presence of more than two alleles per locus has also been observed in other *Phytophthora* species such as *Phytophthora infestans* ^49^, *Phytophthora nicotianae* ^50^ and *P. ramorum* ^51^. The precise mechanism for the observed aneuploidy in *Phytophthora* spp. remains unknown. However, it has been suggested that this was generated by non-Mendelian inheritance during sexual recombination^52^ or by gene duplication^51^.

The intraspecific genetic diversity of *P. cinnamomi* has received a great deal of attention in recent years. The first studies used isozyme analysis to measure genetic diversity of the pathogen^11,12,53^. Results revealed a low genetic diversity among isolates of A2 mating type in contrast to the high diversity of A1 mating type isolates. In another study of a South African population of *P. cinnamomi* nine different multilocus isozyme genotypes were identified^15^, seven of these were A2 mating type and two were A1. In general, these studies indicated a low level of genetic diversity along with a low number of alleles per locus. In addition, no geographic pattern of genotype distribution was observed and it was also suggested that sexual reproduction in natural populations was rare to non-existent. Our data show that *P. cinnamomi* populations from Limpopo and Mpumalanga have a high level of linkage disequilibrium, which is consistent with previous findings^18^, also suggesting that South African populations of *P. cinnamomi* are predominantly under asexual reproduction. This is supported by the absence of the A1 mating type in the Mpumalanga and Limpopo provinces in South Africa.

The South African population of *P. cinnamomi* assessed in this study showed a low level of genotypic diversity for both Shannon-Wiener Index of MLG diversity (G) and Stoddart and Taylor’s Index of MLG diversity (H). A single MLG was present in both populations that accounted for 59% of Limpopo and 55% of Mpumalanga. Nine MLG’s were shared between these two populations with approximately 200 km distance between the two locations. The high level of gene flow (low F_ST_ values) and the presence of multiple shared MLGs suggest that there are frequent migrations between these populations.

The presence of a dominant genotype in Limpopo and Mpumalanga suggests that this genotype may be favourable for the specific environmental conditions. It is therefore critical to include this dominant genotype in screening and selection protocols to identify tolerant or resistant plant material. It is, however, important to keep in mind that population dynamics of pathogens evolve and change over time^54,55^ and constant monitoring of genotypes in a population is necessary to make informed decisions on breeding for resistance and disease management^56^.

In this study, we developed 16 SSR markers for *P. cinnamomi* and evaluated the informativeness of these markers on *P. cinnamomi* populations from South Africa. This highly reproducible and informative set of markers will enable future population studies of *P. cinnamomi*, which will collectively provide better insight into the global genetic diversity and movement of this globally relevant and aggressive pathogen.

## Electronic supplementary material

Supplementary figures
