# Supplementary material for: New microsatellite markers for population studies of Phytophthora cinnamomi, an important global pathogen
Source: Sci Rep. 2017 Dec 15;7:17631. doi: 10.1038/s41598-017-17799-9 (PMC5732169; doi:10.1038/s41598-017-17799-9)
Supplement: Supplementary file 1 — Supplementary figures [file 41598_2017_17799_MOESM1_ESM.doc]

**New microsatellite markers for population studies of *Phytophthora cinnamomi*, an important global pathogen**

J. Engelbrecht^1*^, T.A Duong^2^ and N.v.d Berg^1^

^1^Department of Microbiology and Plant Pathology, ^2^ Department of Genetics, Forestry and Agricultural Biotechnology Institute (FABI) , University of Pretoria, Pretoria 0002, South Africa.

* Corresponding author: Juanita Engelbrecht, Email: Juanita.engelbrecht@fabi.up.ac.za

**Supplementary Figure 1**


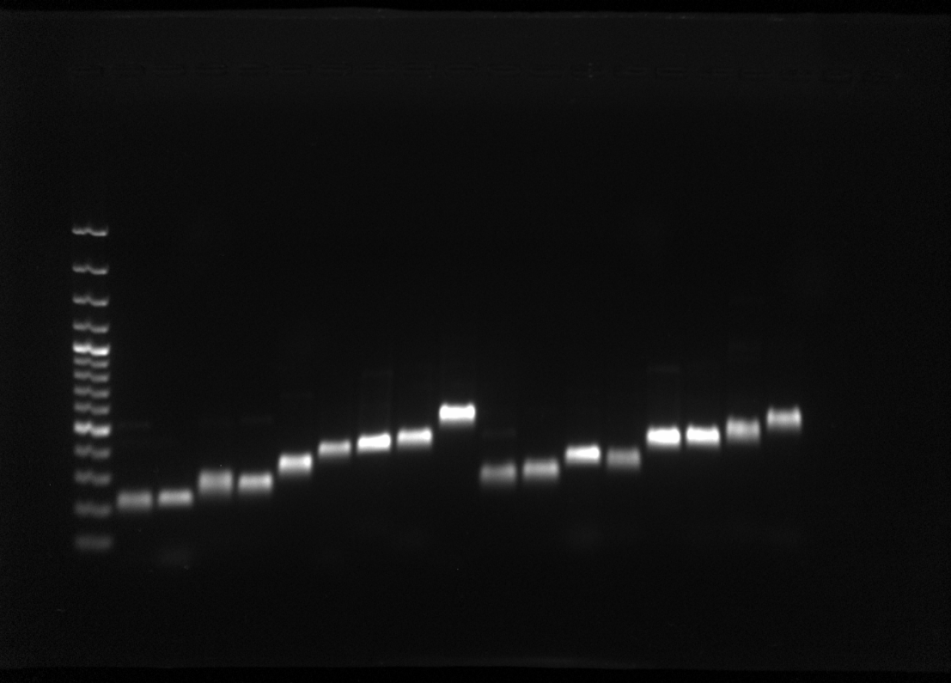


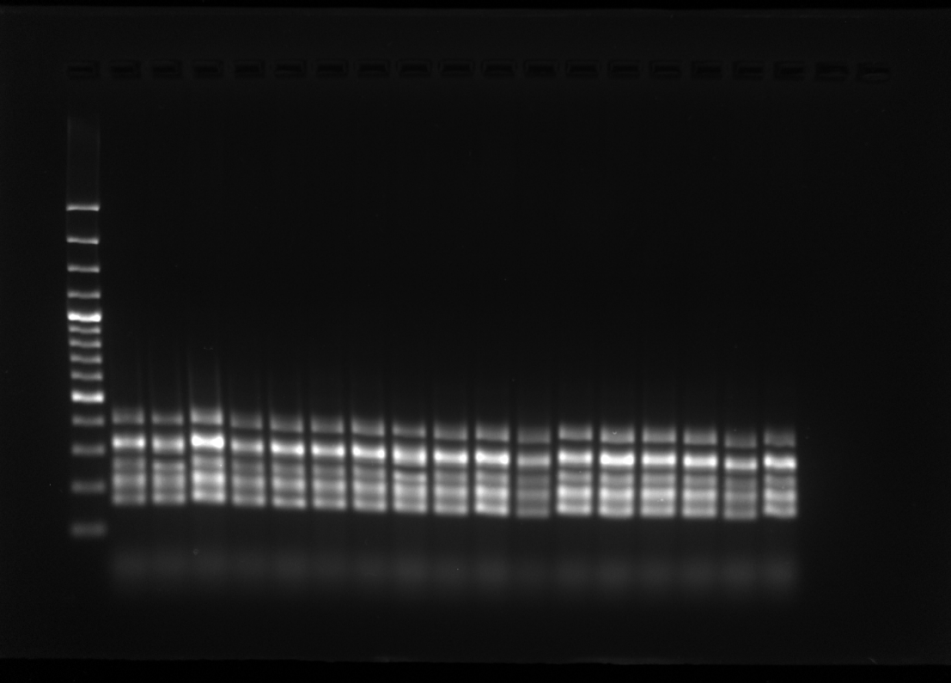


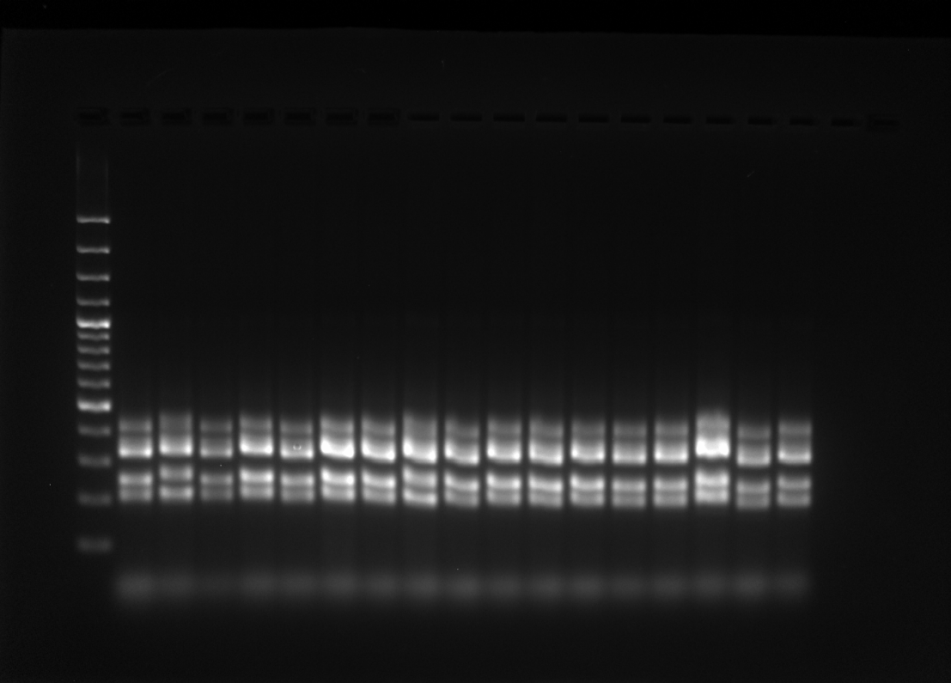


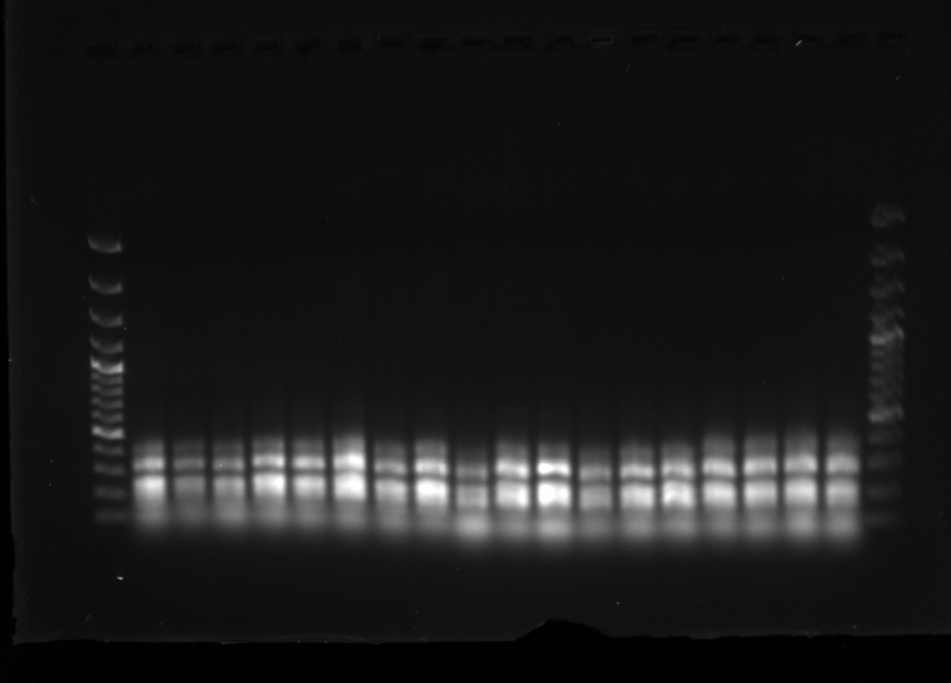


Fig. S1. Original images as per the order they appeared in Fig. 1 in main text. Graphical editing made to these figure to create figure 1 are explained in detail below:

Gel 1: Well 4th (start from the ladder) have been removed as this marker was excluded from this study.

Gel 2: The last well has been removed to fit the figure.

Gel 3: The last well has been removed to fit the figure.

Gel 4: The last three well have been removed to fit the figure.

**Supplementary Figure 2**


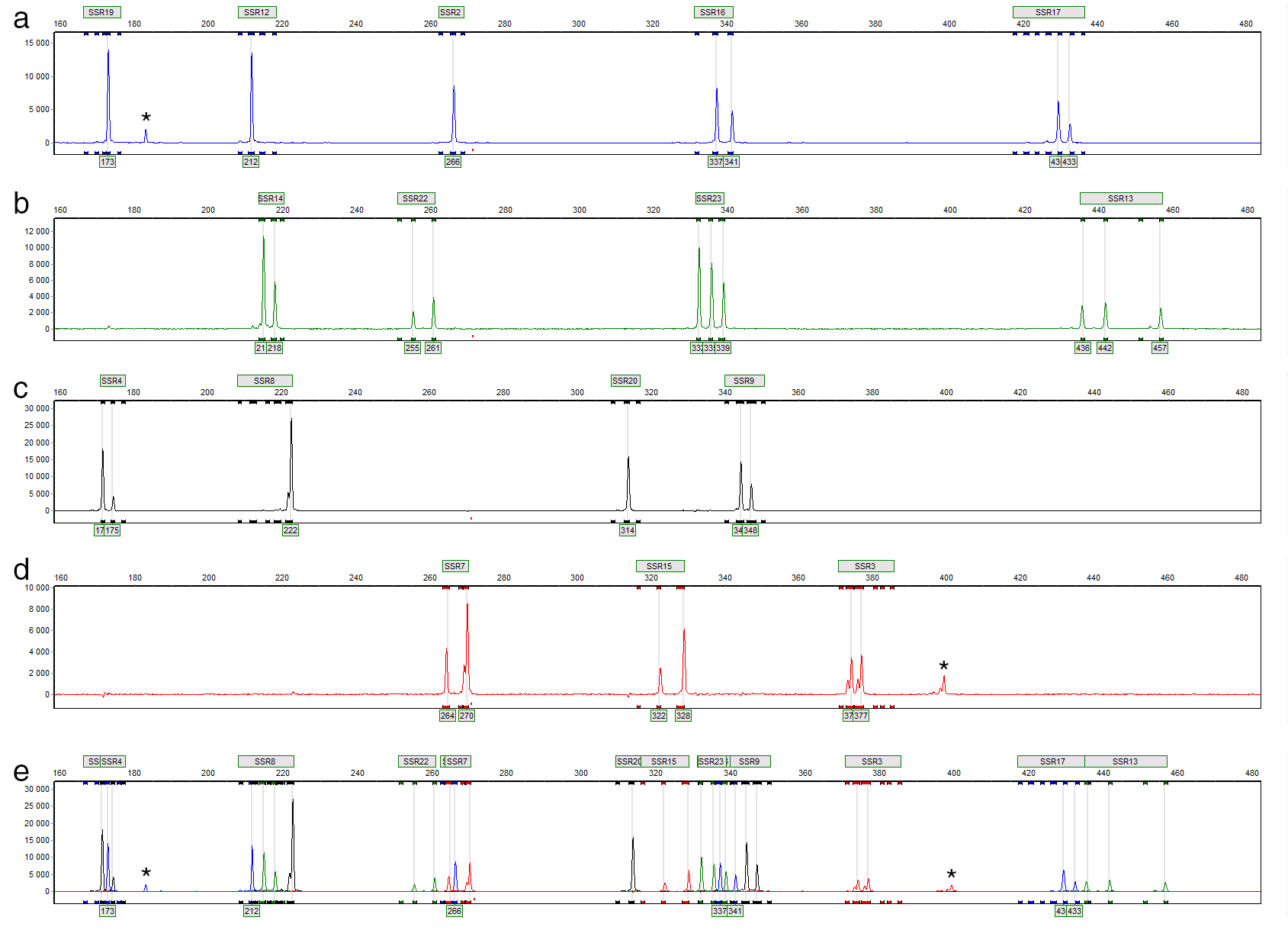


Fig. S2. Electropherograms obtained from GeneScan analysis of multiplex products of 16 markers. a - blue channel (6-FAM); b - green channel (VIC); c - yellow channel (NED); d - red channel (PED); e - all four channels. Asterisks (*) indicate non-specific amplification products derived during multiplex PCR amplification.
